# A Retrospective Study of the Usefulness of Partial Cystectomy for Advanced Colorectal Cancer With Bladder Invasion

**DOI:** 10.7759/cureus.85130

**Published:** 2025-05-31

**Authors:** Toshiyuki Adachi, Yusuke Inoue, Satomi Okada, Takayuki Miyoshi, Akihiko Soyama, Kazuma Kobayashi, Tomohiko Adachi, Kengo Kanetaka, Susumu Eguchi

**Affiliations:** 1 Department of Surgery, Nagasaki University, Nagasaki, JPN

**Keywords:** colorectal cancer, colorectal surgery, locally advanced colon cancer, locally advanced rectal cancer, quality of life (qol)

## Abstract

Background and aim

Bladder invasion in colorectal cancer often necessitates total pelvic exenteration (TPE), a highly radical procedure that offers excellent curability but significantly impairs postoperative quality of life (QoL), especially due to urinary diversion. Although TPE is considered the standard approach, partial cystectomy (PC) may be a feasible option in selected patients. Oncological curability and satisfactory QoL may be achieved with appropriate patient selection. This study aimed to evaluate short-term outcomes and voiding-related QoL in patients with colorectal cancer and bladder invasion who underwent either PC or TPE at a single institution.

Methods

This retrospective study included patients diagnosed with colorectal cancer and bladder invasion between May 2011 and February 2023 who underwent TPE or PC. Perioperative factors such as duration of surgery, blood loss, hospital stay, pathological outcomes, and short-term results were compared. In the PC group, postoperative urinary function was assessed using the Overactive Bladder Symptom Score (OABSS), International Prostate Symptom Score (IPSS), and IPSS-QoL.

Results

Twenty-two patients were included in this study, with eight and 14 patients in the PC and TPE groups, respectively. The duration of surgery and blood loss (p<0.001 and p=0.012, respectively) were lower in the PC group. Pathological curability and recurrence rates were not significantly different. In the PC group, the median pre- and postoperative IPSSs were 1 and 6, respectively; IPSS-QoL was 3.5 postoperatively. The median OABSS increased from 0.5 to 3.5. Median increases in IPSS and OABSS were 3 and 2, respectively.

Conclusion

With appropriate patient selection, PC may provide oncological outcomes comparable to those of TPE while better preserving postoperative QoL in patients with colorectal cancer and bladder invasion.

## Introduction

Colorectal cancer invades the surrounding organs when it progresses. Infiltration into the urinary tract organs within the pelvis is an important factor in the selection of surgical procedures. The surgical procedures for locally advanced colorectal cancer with bladder invasion are colorectal resection with partial cystectomy (PC), preserving the urinary tract, and total pelvic exenteration (TPE), which does not preserve the urinary tract. En bloc resection in locally advanced cancer is important for improving its curative effect, and TPE is an important procedure in expansion surgery for locally advanced colorectal cancer [1-3]. TPE increases the rate of local radical resection because it removes the prostate, seminal vesicles, bladder, large intestine, or rectum with en bloc [4]; however, it requires a change of urinary tract using an ileal conduit, which significantly reduces postoperative patient’s quality of life (QoL) [5,6]. Although PC does not require diversion of the urinary tract and can maintain the patient’s QoL, it may also reduce the degree of cancer curability. Consequently, the extent to which urinary function is maintained after PC remains an important issue. Recent evidence indicates that PC may yield favorable oncologic outcomes when applied to appropriately selected patients [7,8]. In this context, the integration of QoL assessments alongside oncologic evaluation may further underscore the clinical relevance of organ-preserving strategies such as PC. Therefore, this study compared the treatment outcomes of TPE and PC for colorectal cancer with bladder invasion and evaluated urinary function following PC in terms of symptom status and the patient’s QoL using the Overactive Bladder Symptom Score (OABSS), International Prostate Symptom Score (IPSS), and IPSS-QoL score, which are urinary function evaluation scoring sheets useful in urology.

## Materials and methods

Study population

In this retrospective study, we evaluated the data of patients with colorectal cancer who underwent surgery at the Department of Surgery, Nagasaki University Hospital, Nagasaki, Japan, between May 2011 and February 2023. The eligibility criteria were as follows: (1) diagnosis of colorectal cancer using colonoscopy and biopsy, (2) suspected bladder invasion based on preoperative imaging evaluation and a diagnosis of clinical tumor stage 4b, and (3) R0 resection achieved using simultaneous or two-stage resection in stage IV cases. The cancer stage was defined according to the Union for International Cancer Control Guidelines, Eighth Edition. The exclusion criteria were malignancies other than adenocarcinoma on histological examination, hereditary colorectal cancer, and inflammatory bowel disease. Patients who met the eligibility criteria were assigned to either the PC or TPE group. This study was approved by the Nagasaki University Hospital Research Ethics Committee (approval number: 19102141; dated October 21, 2019) and performed in accordance with the principles of the Declaration of Helsinki. All patients were provided information regarding the study and consented to data collection for colorectal cancer research. This observational study was designed and reported in accordance with the Strengthening the Reporting of Observational Studies in Epidemiology statement.

Data collection and evaluation

Age, sex, body mass index (BMI), history of diabetes mellitus and oral steroid use, preoperative tumor marker values, tumor location, preoperative treatment, presence of obstructive symptoms, presence or absence of tumor exposure in the bladder on cystoscopy, and clinical stage were recorded. The perioperative data included the operative time, estimated blood loss, operative procedure, and length of hospital stay. The histological classification, tumor stage, nodal stage, positive proximal margins (PMs), positive distal margins (DMs), positive radial margins (RMs), and postoperative adjuvant chemotherapy rates were recorded. Postoperative events were described using the Clavien-Dindo classification. Complications were graded according to the Clavien-Dindo classification, and the groups were compared on the basis of the number of grade III and IV complications. Preoperative and postoperative voiding function assessments were recorded using the IPSS and OABSS. The OABSS and IPSS questionnaires were administered by attending surgeons either in person or online at three months or later after surgery. In patients with cognitive impairment, the questionnaires were not administered, and these were recorded as missing data.

Statistical analysis

Continuous and categorical data are presented as medians (interquartile range {IQR}) and numbers and percentages, respectively. All statistical analyses were conducted using JMP Pro 16 (Redmond, WA: Microsoft Corp.) for Windows. The chi-square or Fisher's exact test was used to analyze categorical variables. Analysis of Variance was performed to compare three or more groups. The Mann-Whitney U test was used to compare continuous variables. The log-rank test was used to test the Kaplan-Meier curve. Statistical significance was set at p<0.05. No generative AI or AI-assisted technologies were used in the writing, editing, or production of this manuscript. All content was prepared solely by the authors.

## Results

Patient and surgical characteristics

**Figure 1:**
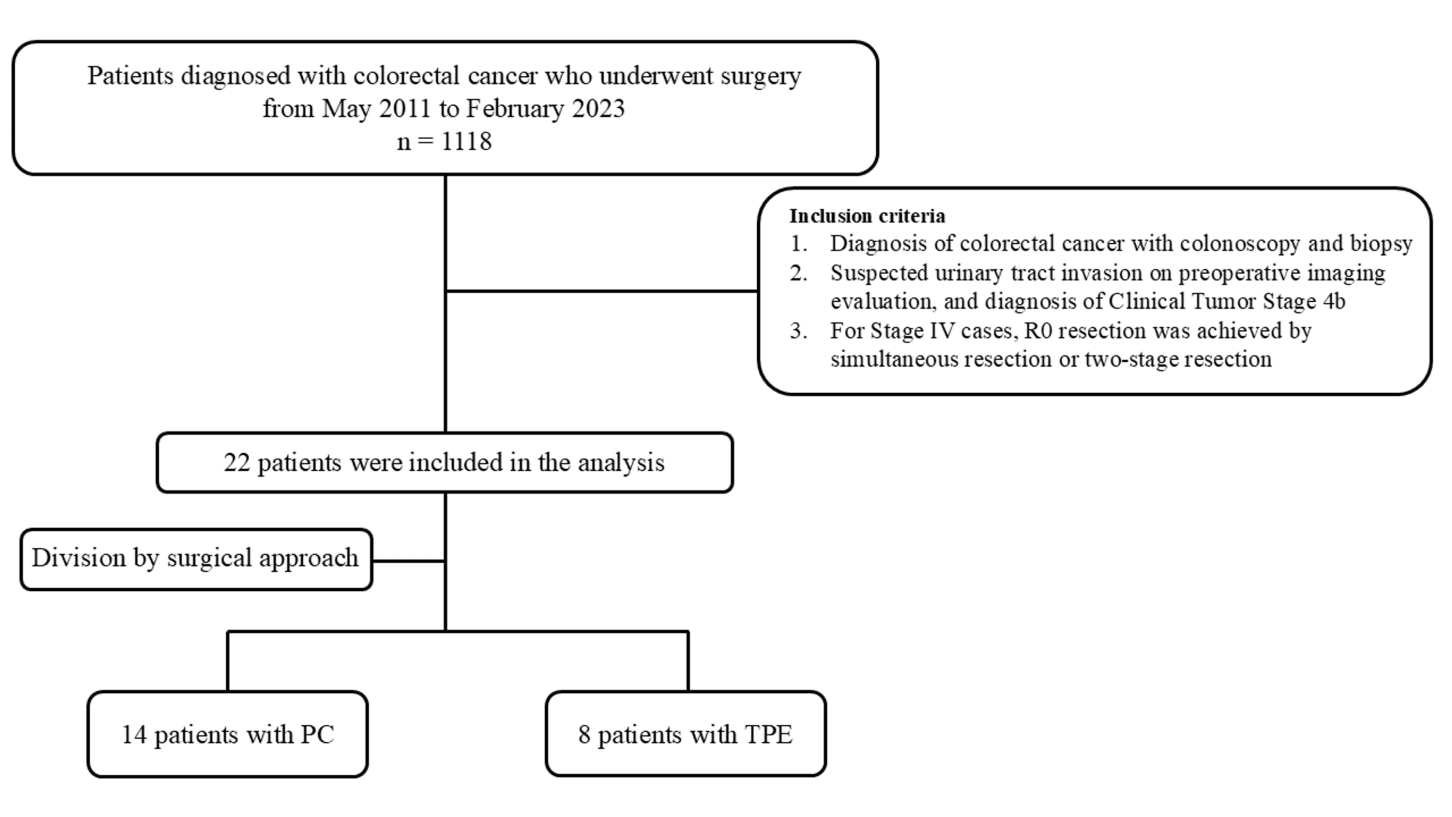
Flow diagram of patients undergoing partial cystectomy and total pelvic exenteration. PC: partial cystectomy; TPE: total pelvic exenteration

A total of 1,118 patients underwent surgical resection for colon cancer at our hospital between May 2011 and February 2023. Twenty-two patients were diagnosed with T4b (bladder) preoperatively, of which 14 and eight underwent PC and TPE, respectively. Patients with visible tumor exposure in the bladder on preoperative cystoscopy underwent TPE, while those without visible tumor exposure and in whom bladder trigone preservation was feasible were selected for PC (Figure 1).

Regarding patient background characteristics, no significant differences were found in age, sex, BMI, presence or absence of diabetes, history of oral steroid use, tumor marker values, tumor localization, rate of preoperative chemotherapy, history of obstructive symptoms, or clinical stage between the groups (Table 1).

**Table 1:** Comparison of demographic and perioperative variables between the partial cystectomy group and the total pelvic exenteration group. *P-values represent overall group comparisons using Analysis of Variance (ANOVA). PC: partial cystectomy; TPE: total pelvic exenteration; BMI: body mass index; DM: diabetes mellitus

Variables	PC (n=14)	TPE (n=8)	p-Value
Age, median (IQR)	73 (63-78)	67 (55-78)	0.412
Sex - female, n (%)	5 (35.7)	2 (25.0)	1.000
BMI, median (IQR)	20.0 (18.2-23.5)	19.9 (18.9-21.4)	0.785
DM, n (%)	4 (28.6)	1 (12.5)	0.613
Steroid use, n (%)	0 (0.0)	0 (0.0)	-
CEA, median (IQR)	4.4 (3.3-7.9)	3.4 (2.2-13.7)	0.245
CA19-9, median (IQR)	14.1 (8.0-38.5)	17.8 (8.6-85.1)	0.847
Tumor location - sigmoid colon	8 (57.1)	3 (37.5)	0.116*
Tumor location - upper rectum	4 (28.6)	2 (25.0)	-
Tumor location - middle rectum	2 (14.3)	0 (0.0)	-
Tumor location - low rectum	0 (0.0)	3 (37.5)	-
Preoperative chemotherapy, n (%)	8 (57.1)	7 (87.5)	0.193
Obstruction, n (%)	11 (78.6)	6 (75.0)	1.000
Presence of tumor exposure in the bladder on cystoscopy	0 (0)	8 (100)	<0.0001
cT stage 1, n (%)	0 (0.0)	0 (0.0)	0.779*
cT stage 2, n (%)	5 (35.7)	3 (37.5)	-
cT stage 3, n (%)	7 (50.0)	3 (37.5)	-
cT stage 4, n (%)	2 (14.3)	2 (25.0)	-

Surgical short-term outcomes and pathological findings

The median operative time was significantly shorter in the PC group than in the TPE group (391 vs. 791.5 min, p<0.001). The median estimated blood loss was significantly lower in the PC group than in the TPE group (202 vs. 1,347 mL, p<0.012). Regarding the surgical approach, no significant difference was found between the open and laparoscopic approaches in either group (65.4% and 37.5%, respectively; p=0.378). The incidence of Clavien-Dindo grade ≥III complications did not significantly differ between the two groups (28.6% and 12.5%, respectively; p=0.613) (Table 2). Regarding pathological factors, the TPE group had significantly more T4b cases than the PC group. The pathological curability did not significantly differ between the two groups.

**Table 2:** Surgical short-term outcomes and pathological findings. *P-values represent overall group comparisons using Analysis of Variance (ANOVA). DM: distal margin; HAR: high anterior resection; lap: laparoscopic; tub: tubular adenocarcinoma; LAR: low anterior resection; muc: mucinous adenocarcinoma; pap: papillary adenocarcinoma; PM: proximal margin, pN stage: pathological nodal stage; por: poorly differentiated adenocarcinoma; p stage: pathological stage; pT stage: pathological tumor stage; RM: radial margin; PC: partial cystectomy; TPE: total pelvic exenteration

Variables	PC (n=14)	TPE (n=8)	p-Value
Type of procedure - sigmoidectomy	8 (57.1)	-	0.378*
Type of procedure - HAR	3 (21.4)	-	-
Type of procedure - LAR	2 (14.3)	-	-
Type of procedure - Hartmann	1 (7.2)	-	-
Approach - laparoscopic	9 (65.4)	3 (37.5)	0.378
Operation time, median (IQR)	391 (334.5-460)	791.5 (543.5-860)	<0.001
Blood loss, median (IQR)	202 (49.5-830.75)	1347 (556.7-3831.2)	0.012
Clavien-Dindo classification >III, n (%)	4 (28.6)	1 (12.5)	0.613
Adjuvant chemotherapy, n (%)	11 (78.6)	6 (75.0)	1.000
Postoperative hospital stay, days	24.5 (14-32)	29 (24-35)	0.273
Historical type - tub	13 (92.8)	6 (75.0)	0.544*
Historical type - por	0 (0.0)	1 (12.5)	-
Historical type - muc	1 (7.2)	1 (12.5)	-
Historical type - pap	0 (0.0)	0 (0.0)	-
pT stage 1	1 (7.2)	0 (0.0)	0.034*
pT stage 2	1 (7.2)	0 (0.0)	-
pT stage 3	7 (50.0)	0 (0.0)	-
pT stage 4	5 (35.6)	8 (100)	-
pN stage 0	10 (71.4)	4 (50.0)	0.274*
pN stage 1	4 (28.6)	2 (25.0)	-
pN stage 2	-	1 (12.5)	-
pN stage 3	-	1 (12.5)	-
p stage 1	2 (14.3)	0 (0.0)	0.779*
p stage 2	6 (42.0)	4 (50.0)	-
p stage 3	5 (36.5)	3 (37.5)	-
p stage 4	1 (7.2)	1 (12.5)	-
PM positive, n (%)	0 (0.0)	0 (0.0)	-
DM positive, n (%)	0 (0.0)	0 (0.0)	-
RM positive, n (%)	0 (0.0)	1 (12.5)	1.000

Postoperative recurrence type and recurrence-free survival

Postoperative recurrence was observed in three (21.4%) and two (25.0%) patients in the PC and TPE groups, respectively, without a significant difference (p=1.00). The type of recurrence was distant metastasis in all three patients in the PC group (two lung metastases and one liver metastasis). In contrast, one patient in the TPE group had pelvic recurrence (Table 3).

**Table 3:** Postoperative recurrence rate and location of recurrence. PC: partial cystectomy; TPE: total pelvic exenteration

Variables	PC (n=14)	TPE (n=8)	p-Value
Recurrence, n (%)	3 (21.4)	2 (25.0)	1
Local, n (%)	0 (0)	1 (50.0)	-
Distant, n (%)	3 (100)	1 (50.0)	-

**Figure 2:**
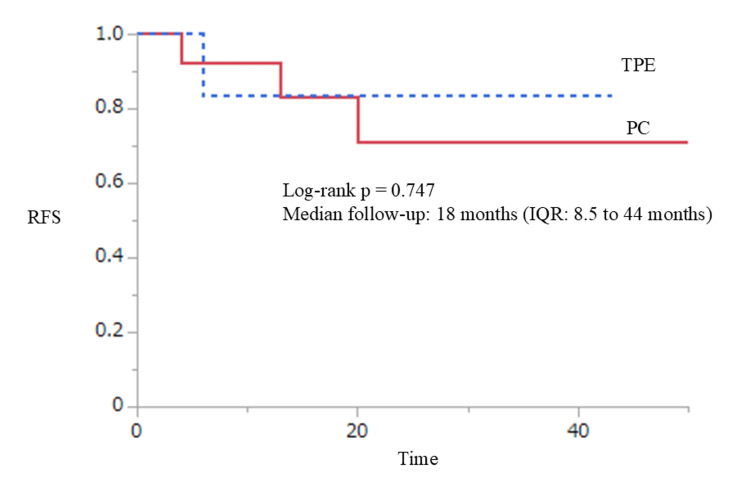
Recurrence-free survival following PC and TPE. PC: partial cystectomy; TPE: total pelvic exenteration

No significant difference was found in recurrence-free survival time between the two groups (log-rank p=0.747). The median follow-up was 18 (IQR: 8.5-44) months (Figure 2).

Evaluation of urinary function satisfaction following PC

**Figure 3:**
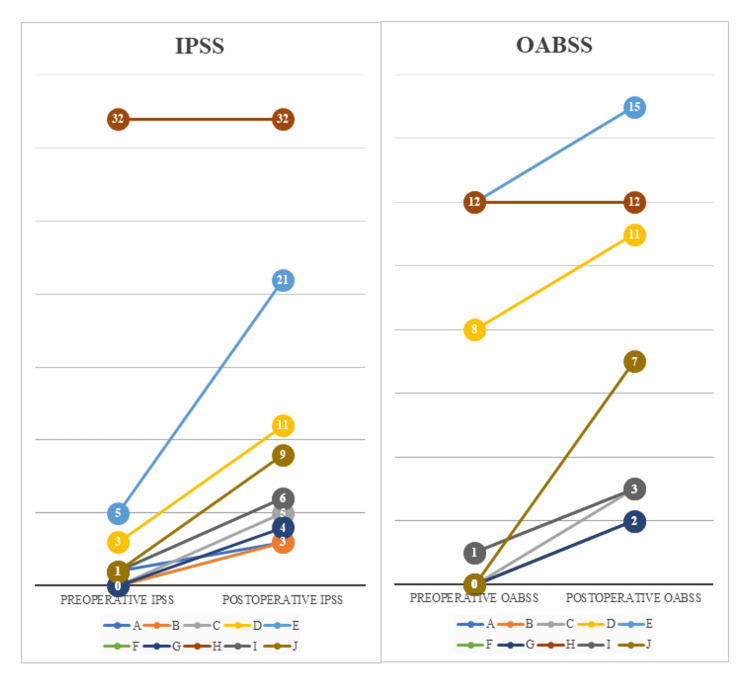
A line graph illustrating the changes in IPSS and OABSS before and after PC, highlighting the rate of increase for each individual. A-J represent the individuals. IPSS: International Prostate Symptom Score; OABSS: Overactive Bladder Symptom Score

Urinary function following PC was evaluated using the IPSS, IPSS-QoL, and OABSS. The median IPSSs before and after PC were 1 (0-32) and 6 (3-32), respectively. The median IPSS-QoL after PC was 3.5 (2-6), the median OABSS before PC was 0.5 (0-12), and the median OABSS score after PC was 3.5 (2-15). The median increases in IPSS and OABSS postoperatively were 3 (0-16) and 2 (0-7), respectively (Table 4 and Figure 3).

**Table 4:** Preoperative and postoperative IPSS and OABSS scores. IPSS: International Prostate Symptom Score; OABSS: Overactive Bladder Symptom Score; QoL: quality of life

Variables	Preoperative (n=10)	Postoperative (n=10)	Increases in score
IPSS	1 (0-32)	6 (3-32)	3 (0-16)
IPSS-QoL score	-	3.5 (2-6)	-
OABSS	0.5 (0-12)	3.5 (2-15)	2 (0-7)

## Discussion

This study examined the outcomes of PC in treating colorectal cancer with bladder invasion compared with TPE and investigated the QoL in postoperative voiding function with PC to demonstrate the high curative potential of PC and the maintenance of QoL in voiding function. Our findings indicate that if appropriate cases are selected, PC is a useful technique that allows for urinary tract preservation and guarantees a cure without lowering the patient’s QoL.

The pelvis, where the rectum is located, contains several organs in a narrow space. Advanced rectal cancer usually invades surrounding organs, necessitating combined resection. Extended surgery is necessary to perform R0 resection in colorectal cancer with invasion of other organs [9,10].

Colorectal cancer with urinary tract organ involvement, including the bladder, requires extensive surgery with alterations in the urinary tract. TPE was first reported by Brunschwig in 1948 as a useful technique for treating advanced rectal cancer [11]. Although TPE is a highly curative and useful surgical procedure for locally advanced colorectal cancer [12], it is highly invasive and reduces patients’ QoL [13-15].

**Figure 4:**
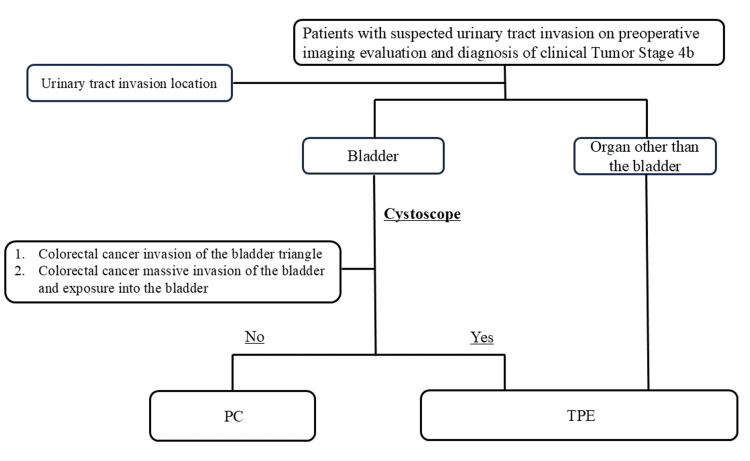
Flow chart of treatment for patients with clinical stage 4b tumors at our institution. PC: partial cystectomy; TPE: total pelvic exenteration

PC is also the procedure of choice for colorectal cancer with bladder invasion in the absence of bladder triangle invasion or massive exposure to the bladder and can preserve the urinary tract but may be less curative than extended resection. However, only a few studies have compared the postoperative outcomes of TPE, extended surgery, and reduced surgery (PC) for the treatment of colorectal cancer with bladder invasion. In the absence of bias in patient background characteristics between the two groups, the positivity rates for PM, DM, and RM (PM: 0% and 0%, DM: 0% and 0%, RM: 0% and 12.5%, respectively) were comparable in terms of pathological curability between PC and TPE. Appropriate patient selection is the first step to ensure the effectiveness of PC. At our facility, we created a flowchart for locally advanced colorectal cancer suspected as stage T4b and used it to help determine patients’ treatment plans (Figure 4).

**Figure 5:**
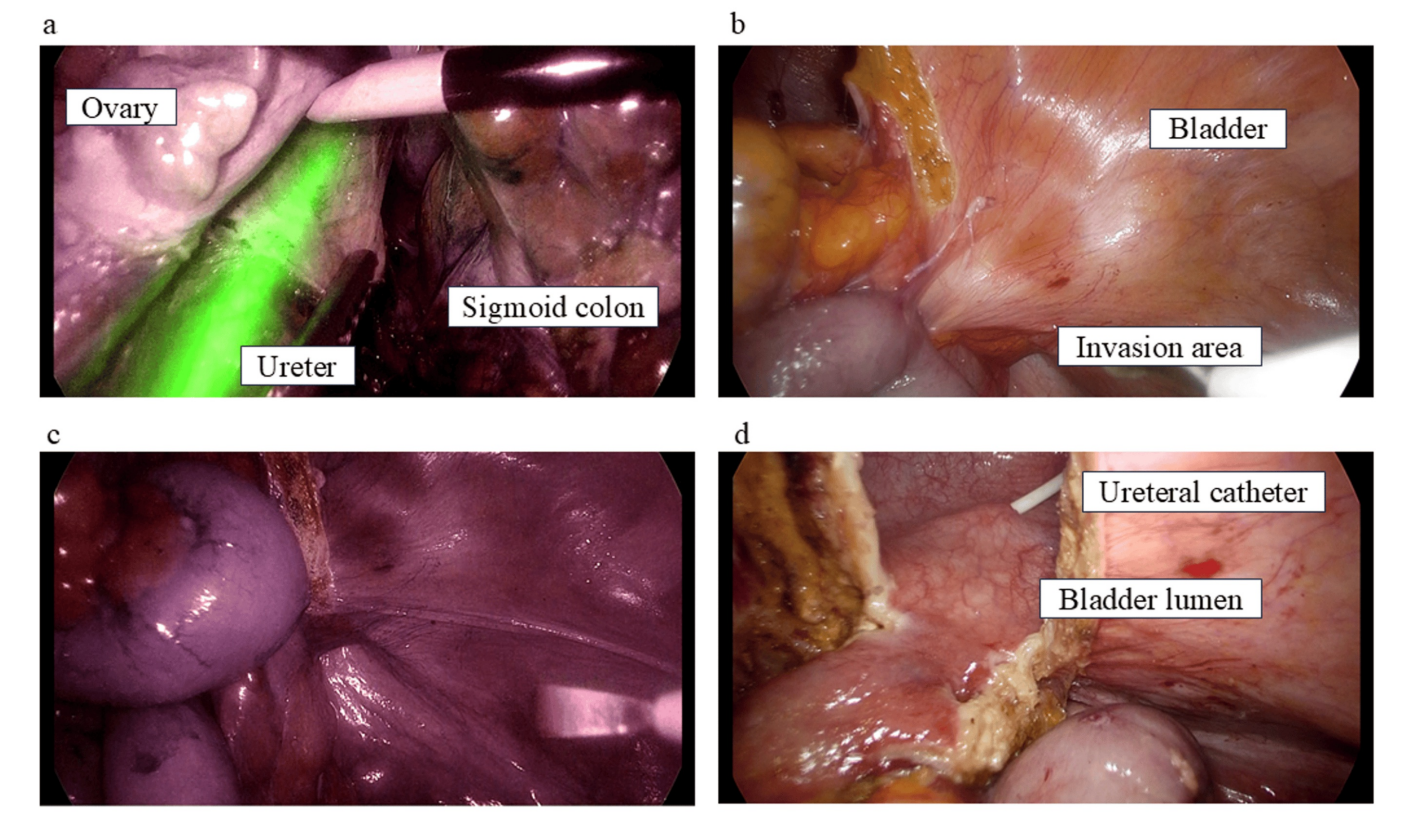
Representative intraoperative image showing partial cystectomy performed with a fluorescent ureteral stent. Placement of the fluorescent stent enabled clear visualization of the ureteral course, allowing for the secure acquisition of an adequate tumor-free margin without causing ureteral injury. (A) Visualization of the ureter with the near-infrared ray catheter. (B) Bladder invasion area of colorectal cancer. (C) Confirmation of the location of the bladder invasion area of colorectal cancer and ureter (confirmation of the lack of a ureter in the planned incision line). (C) PC with a sufficient resection margin. PC: partial cystectomy

The flowchart uses computed tomography and magnetic resonance imaging (MRI) to evaluate urinary tract invasion of colorectal cancer. In our hospital, rectal resection with combined resection of the prostate and seminal vesicles is not an option. TPE is selected in favor of a radical cure in such cases. In bladder invasion cases, cystoscopy is mandatory when evaluated as cT4b. Imaging evaluation is also important for bladder preservation in cases of bladder invasion by colorectal cancer. MRI is useful in assessing the local progression of colorectal cancer [16-19]. However, the concordance rate between MRI and pathological evaluation is imperfect; therefore, a more definitive evaluation of bladder infiltration is required. In this study, 64.4% of patients with PC had invasion depths below T3. In our department, we perform cystoscopy on all patients suspected of having bladder infiltration to accurately determine the T stage. However, massive bladder invasion and tumor exposure in the bladder should not be considered because of the risk of local bladder recurrence [20]. In cases where colorectal cancer exhibits extensive invasion into the bladder, achieving an adequate surgical margin for curative resection necessitates dissection in proximity to the bladder trigone. Preoperative cystoscopic evaluation is highly effective in determining whether bladder preservation is feasible with an appropriate resection line. The second step to ensure a cure for PC is to devise appropriate surgical techniques. Recently, laparoscopic fluorescence navigation surgery has progressed and is being applied in various fields [21-23]. The near-infrared ray catheter (Dublin, OH: Cardinal Health) has enabled determining the course of the ureter during surgery using a laparoscopic camera in near-infrared light mode [24]. Consequently, this has made performing a highly curable PC with a reliable margin possible, even near the bladder triangle (Figures 5a-5d).

The incidence of postoperative complications of Clavien-Dindo IIIa or higher was 28.6% (four cases) in the PC group, which tended to be higher than that in the TPE group. The complications consisted of two cases of anastomotic leakage, one case of deep surgical site infection, and one case of hydronephrosis. The anastomotic leakages did not require emergency surgery and were improved conservatively with drain replacement only. As for hydronephrosis, a ureteral stent was required owing to urinary dysfunction after cystectomy, but it was able to be removed one month later. PC is significantly less invasive than TPE, but during patients’ postoperative clinical course, caution is required regarding the occurrence of urinary tract-related complications associated with bladder preservation and suture leakage associated with inadequate preparatory procedures.

One of the objectives in selecting PC as a reduction surgery is to maintain a patient’s QoL. Urinary tract diversion is necessary for TPE, and many patients have a double stoma. However, no previous studies have focused on QoL assessment of urinary function in patients who have undergone PC. In this study, we used the IPSS, IPSS-QoL, and OABSS, which are applied in urology to assess QoL pertaining to urinary function. The OABSS evaluates postoperative urinary function by scoring the frequency of urination associated with bladder shrinkage. Urinary drainage capacity was evaluated using IPSS. As shown in Table 4 and Figure 2, the median value after PC was 6, which appears to indicate a slight decrease in voiding power, whereas the median rate of increase was 3, suggesting an age-appropriate decline in urinary function preoperatively and that surgery has minimal impact on this decline. In the IPSS-QoL, patients rated their satisfaction with urinary function after PC, with a median score of 3.5, indicating a good impression. The median postoperative OABSS was 3.5, and the median increase due to surgery was 2; therefore, PC is unlikely to cause urinary urgency that would reduce QoL.

This study has some limitations. First, the sample size was small and derived from a single institution, which may limit the ability to detect statistically significant differences, particularly for subtle effects. Although the number of cases of colorectal cancer with bladder invasion is small, the fact that the data were from a single institution with a unified treatment strategy makes this study significant. Second, the observation period was short; therefore, longer monitoring of the local controllability of PC is recommended. Third, this study did not include cystoscopic re-evaluation after preoperative treatment, which limits the assessment of the potential to expand indications for partial cystectomy (PC). Whether PC could be appropriately selected in cases where tumor exposure disappears following preoperative therapy remains an important question that should be addressed in future studies with larger cohorts. Fourth, the OABSS and IPSS evaluations are somewhat subjective data; therefore, to make them more objective, quantifying the voiding function, such as by measuring urinary flow velocity, may be necessary. However, objective data on postoperative voiding function may be inconsistent with a patient’s postoperative QoL, and subjective data on voiding function are important for assessing QoL.

## Conclusions

Our study demonstrated the oncological feasibility of PC in the treatment of colorectal cancer with bladder invasion and its potential to maintain postoperative patient QoL. Furthermore, appropriate patient selection is important when selecting PC, and the long-term course of PC should be carefully monitored.
